# Formation of Indium-Doped Zinc Oxide Thin Films Using Ultrasonic Spray Pyrolysis: The Importance of the Water Content in the Aerosol Solution and the Substrate Temperature for Enhancing Electrical Transport

**DOI:** 10.3390/ma5030432

**Published:** 2012-03-12

**Authors:** Rajesh Biswal, Luis Castañeda, Rosario Moctezuma, Jaime Vega-Pérez, María De La Luz Olvera, Arturo Maldonado

**Affiliations:** 1 Departamento de Ingeniería Eléctrica, Centro de Investigación y de Estudios Avanzados del Instituto Politécnico Nacional CINVESTAV-IPN, SEES, Apartado 14740, México; E-Mails: rroshan@cinvestav.mx (R.B.); molvera@cinvestav.mx (M.L.O.); amaldo@cinvestav.mx (A.M.); 2 Instituto de Física, Benemérita Universidad Autónoma de Puebla, Apartado Postal J-48, Puebla 72570, Mexico; 3 Instituto de Ciencias Básicas e Ingeniería de la Universidad Autónoma del Estado de Hidalgo-AAMF, Pachuca 42184, México; E-Mail: rosario.moctezuma@gmail.com; 4 Escuela Superior de Ingeniería Mecánica y Eléctrica Unidad Ticoman del Instituto Politécnico Nacional, Apartado Postal 07340, D.F., México; E-Mail: jvega@ipn.mx

**Keywords:** zinc oxide, thin solid films, ultrasonic spray pyrolysis, 68.55.-a, 68.55.Jk, 81.10.Dn, 81.15.Rs

## Abstract

Indium doped zinc oxide [ZnO:In] thin films have been deposited at 430°C on soda-lime glass substrates by the chemical spray technique, starting from zinc acetate and indium acetate. Pulverization of the solution was done by ultrasonic excitation. The variations in the electrical, structural, optical, and morphological characteristics of ZnO:In thin films, as a function of both the water content in the starting solution and the substrate temperature, were studied. The electrical resistivity of ZnO:In thin films is not significantly affected with the increase in the water content, up to 200 mL/L; further increase in water content causes an increase in the resistivity of the films. All films show a polycrystalline character, fitting well with the hexagonal ZnO wurtzite-type structure. No preferential growth in samples deposited with the lowest water content was observed, whereas an increase in water content gave rise to a (002) growth. The surface morphology of the films shows a consistency with structure results, as non-geometrical shaped round grains were observed in the case of films deposited with the lowest water content, whereas hexagonal slices, with a wide size distribution were observed in the other cases. In addition, films deposited with the highest water content show a narrow size distribution.

## 1. Introduction

Zinc oxide (ZnO) in thin film form shows a wide variety of controlled properties, such as piezoelectricity, gas chemisorption, photo-catalysis, high conductivity, and transparency in the visible spectrum, that can be exploited in the design and manufacture of optoelectronic and electronic devices as well as in catalysis and biology fields [1,2,3].

According to the required application, the deposition of ZnO thin films can be done successfully by different physical techniques, as is the case of sputtering [4], reactive evaporation [5], pulsed laser deposition [6], and chemical techniques—sol-gel [7], chemical bath [8], chemical vapor deposition (CVD) [9], and chemical spray (CST) [10]. Among these deposition techniques, CST has been refined over the years for industrial applications due to the direct implementation of large area deposition using low cost equipment [11]. Additionally, for transparent conductors, chemically sprayed as-grown films do not require an extra annealing step and it can also be considered as a key technique for future developments in nano-scale manufacturing [12].

Focusing only on CST, there have been extensive studies on the effect of deposition variables on the physical characteristics of ZnO thin films [13,14,15,16,17,18]. However, all this time CST has not remained the same, since substantial modifications in the set up have been successfully tried in order to enhance the characteristics of the films. The atomization process can play a key role in the deposition of ZnO thin films with high transmittance and conductivity, as well as textured surface, adequate for transparent electrodes in thin film solar cells. As a matter of fact, the ultrasonic atomization process generates smaller droplets than the conventional pneumatic process, which in turn yield films with a smoother surface and enhanced conductivity.

The effect of deposition variables on the characteristics of undoped [19,20] and doped ZnO films [21,22,23,24,25,26,27,28,29] deposited with ultrasonic spray pyrolysis has been reported. It is worthy to note that the number of reports of ZnO thin films based on ultrasonic process is increasing, although not yet at the same depth of knowledge than those based on pneumatic process. In this respect, some home-made adapted equipment, including commercial nebulizers, have been used for depositing conductive and transparent ZnO thin films, as is noted in different reports. Unfortunately, this influences the number of reports regarding ZnO dependence on deposition variables. Therefore, a lack of knowledge about some deposition variables, affecting the performance of ZnO thin films as transparent and conductive materials is still present in the literature. The effect of solvent composition, which is relevant in the deposition process as the gaseous species formed affect the final structure of films, has been pointed out previously by Smith and Rodriguez Clemente [30]. Finding some correlation between deposition conditions and physical characteristics of the ZnO thin films was expected. The main goal of our study was the optimization of the deposition conditions in order to manufacture highly conductive and transparent ZnO thin films on glass substrates by ultrasonic chemical spray, avoiding extra steps such as a previous synthesis, complex reactor design and annealing on vacuum, or reducing atmospheres.

In this work the effect of water content variation in the starting solution on the electrical, structural, morphological, and optical characteristics of indium doped zinc oxide thin films, deposited by chemical spray at 430°C, is studied.

## 2. Experimental Details

### 2.1. Fabrication Of Indium Doped Zinc Oxide [ZnO:In] Thin Films

**Figure 1:**
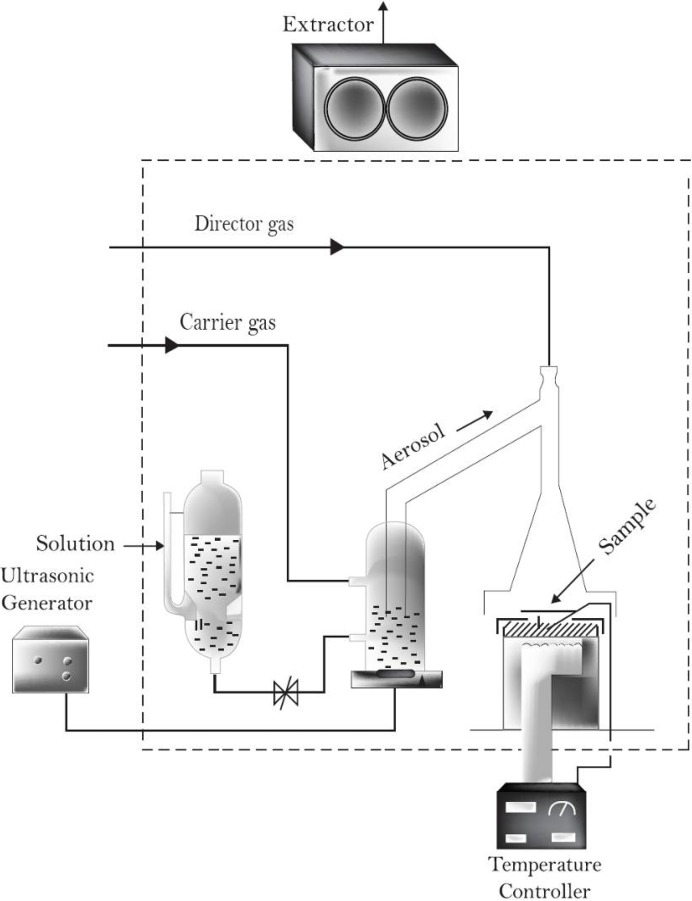
Schematic diagram of the experimental set up used for depositing the ZnO:In thin films (ultrasonic spray pyrolysis: USP).

ZnO:In thin solid films were prepared using the ultrasonic spray pyrolysis (USP) technique (Figure 1), which is a versatile technique that can be used to produce nanoscale sized powders and thin solid films. With this method the particle’s size can be easily controlled by changing the concentration in the starting solution and the atomization parameters. The deposition system used for depositing the ZnO:In thin films presented in this work includes a piezoelectric transducer operating at variable frequency, which was set to 1.2 MHz and the ultrasonic power at 120 W.

ZnO:In thin films were deposited on 2.54 cm × 2.54 cm soda-lime glass substrates from five different starting solutions. The starting solutions were prepared from a 0.2 M solution of zinc (II) acetate ([Zn(O_2_CCH_3_)_2_] from Alfa, 98%), dissolved in a mix of deionized water, acetic acid ([CH_3_CO_2_H] from Baker, 95%), and methanol ([CH_3_OH] from Baker, 98%). For a 1 L solution the acetic acid quantity was fixed to 50 mL/L in all cases. Separately, a 0.2 M solution of indium (III) acetate ([In(CH_3_CO_2_)_3_] from Alfa, 98 %) dissolved in a mix of deionized water and acetic acid (1:1, volume proportion) was prepared in order to be used as doping solution. A constant [In]/[Zn] ratio of 3.0 at.% was used. The five starting solutions were prepared with different water contents, namely, (M1) 50 mL/L, (M2) 100 mL/L, (M3) 150 mL/L, (M4) 200 mL/L, and (M5) 300 mL/L. Finally methanol was added until 1 L was completed. The substrates were cleaned prior to deposition. The cleaning process of the substrates is as follows: (i) sonication for five minutes in trichloroethylene ([C_2_HCl_3_] from Baker, 98%) for degreasing the substrates; followed by (ii) sonication in methyl alcohol ([CH_3_OH] from Aldrich, 98%); (iii) sonication in acetone ([CH_3_COCH_3_] from Baker, 98%), and finally, (iv) the substrates are dried by a jet of pure and dry nitrogen ([N_2_] from PRAXAIR, 99.997%). Then the substrates are placed on a fused tin bath, whose temperature measured just below the substrate using a chromel-alumel thermocouple, which is contained in a stainless steel metal jacket. The substrate temperature (*T_s_*) was kept constant at 430 °C, within an accuracy of ±0.5 °C. Pure N_2_ (from PRAXAIR, 99.997%) was used as solution carrier and director gas, with flow rates of 3.5 and 0.5 L·min^−1^, respectively.

### 2.2. Indium-Doped Zinc Oxide Thin Solids Films Analysis

The thickness of the ZnO:In thin films was measured by a KLA profilometer (Tencor model P15 with a resolution of 1.5 nm) on a step formed during deposition. All the samples were grown with a film thickness value around 800 nm. The microcrystalline structure was studied from X-ray diffraction analysis of the samples made in a Siemens D5000 diffractometer by using the Cu-K_1_ (λ = 0.154056 nm) radiation and the θ-2θ technique. A JEOL scanning electron microscope (SEM) was used for morphological and composition evaluation of the thin solid films. The optical transmittance spectra at normal incidence were obtained by a double-beam Shimadzu 2401 PC spectrophotometer, in the UV-Vis region (350–1000 nm) without glass substrate correction. Electrical sheet resistance of samples was measured by the conventional four-aligned probe method (Veeco equipment) with the appropriate geometric correction factors.

## 3. Results and Discussion

### 3.1. Structural Properties

**Figure 2:**
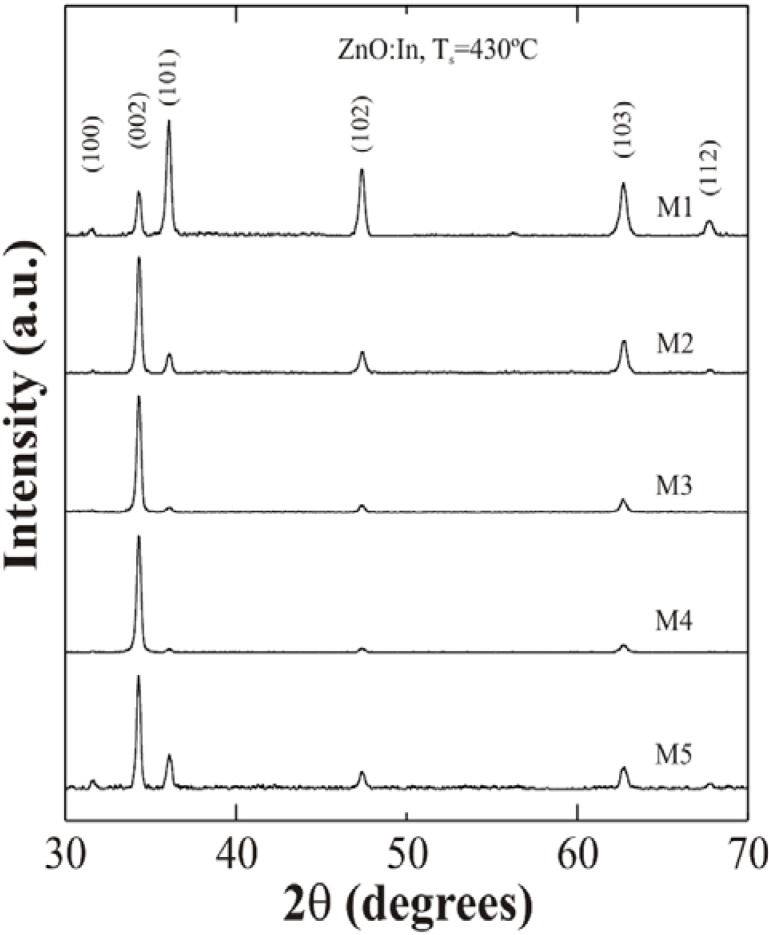
X-ray diffraction patterns of ZnO:In thin films deposited at 430 °C from the starting solutions with different water content.

X-ray diffraction patterns obtained from ZnO thin films show polycrystalline characteristics and the corresponding peaks fit well to a hexagonal ZnO wurtzite type structure in all cases. However, changes in the intensity of the peaks were observed according to the deposition conditions. Figure 2 shows the patterns of ZnO:In thin films deposited at 430 °C with different water contents in the starting solution. It can be seen that the sample deposited with the lowest water quantity does not shows a preferential growth with respect to the low order reflections. However, when water content increases to 100 mL/L, the corresponding spectrum of ZnO:In films shows the (002) intensity prevailing over all the rest. High order reflections exhibit only a marginal contribution. This situation is presented in all remaining cases.

The crystallite size was estimated using the (002) and (100) diffraction peaks from the XRD data in accordance with the Debye-Scherer formula [31]:

$$D=\frac{0.9\;\lambda }{B\;\cos\theta }$$

where *D* is the crystallite size in nanometers, *λ* is the wavelength value of the Cu *K_α1_* line, *θ* is the Bragg diffraction angle and *B* is the FWHM of the diffraction peak measured in radians. According to these results, the crystallite size of the ZnO:In thin films decreases from 36 to 23 nm as the deionized water concentration is increased in the starting solution.

### 3.2. Scanning Electron Microscopy Studies

**Figure 3:**
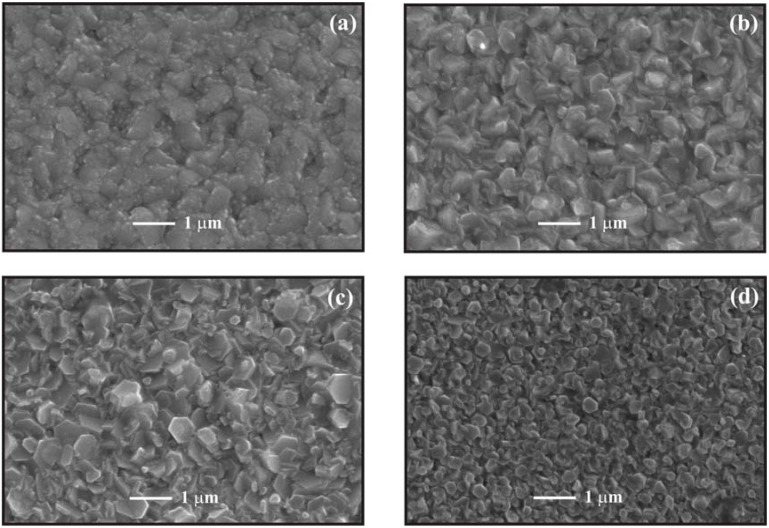
SEM micrographs of ZnO:In thin films deposited at 430 °C from starting solutions with different water contents: (**a**) 50 mL/L, (**b**) 100 mL/L, (**c**) 200 mL/L, and (**d**) 300 mL/L.

Figure 3(a) shows the surface morphology of ZnO:In thin films deposited at 430 °C from different water contents in the starting solution. In the case of films deposited with 50 mL/L, two grain sizes; round shaped grains with an average size of 120 nm, and others not well defined, elongated and bigger, with an average size of 1 μm, were observed. At higher zoom it is possible to note that the surface of these grains is covered by an agglomeration of smaller grains. The films deposited from a solution with water content of 100 mL/L show a dramatic change, owing to the fact that at this stage the grains present a well defined shape, namely hexagonal slices with a wide size distribution, as it can be seen in Figure 3(b). Size distribution ranging from 150 to 800 nm was observed. In addition, stacking of hexagonal slices was also observed. Figure 3(c) shows the morphology of samples deposited with a water content of 200 mL/L in the starting solution; a decrease in the fraction of smaller grains, formed by hexagonal shaped thin slices, is observed as well as a decrease in the size of the bigger grains. In fact, the maximum size is now in the order of 700 nm. Figure 3(d) shows the morphology of ZnO:In thin films deposited with the highest water content, namely 300 mL/L. Hexagonal slices can again be observed, but now with a uniform grain size, in the order of 350 nm. A small fraction, in the order of 10% is formed by grains with a smaller size. No stacking of hexagonal slices was observed in this case. It should be noted that as the water content increases, the grain size of ZnO samples, measured in the SEM micrographs, decreases; whereas the corresponding crystallite size, estimated from X ray diffraction data, shows a marginal increase. The effect of water content on the morphology and distribution grain size of ZnO:In thin films is apparent.

### 3.3. Optical Properties

**Figure 4:**
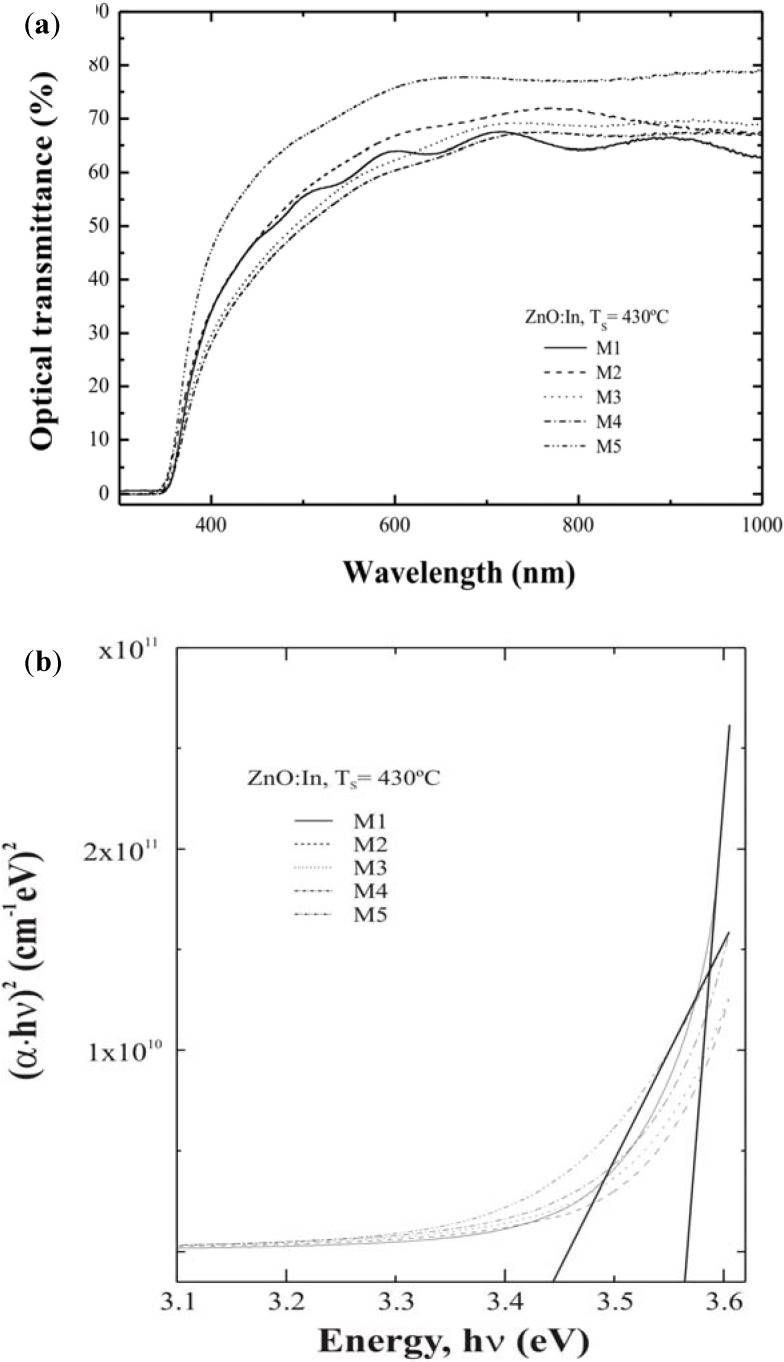
(**a**) Optical transmittance spectra and (**b**) Plot of (αhν)^2^ *vs.* hν.

Figure 4(a) shows the optical transmittance spectra of ZnO thin films deposited from starting solutions with different water contents. No substrate correction was made in the corresponding measurement. Average transmittance of all the films is suited for TCO application. In Table 1, the corresponding film thickness for every sample is shown. Optical band gap was calculated from the plot of ${\left(\alpha h\nu \right)}^{2}$ as a function of *hν*, where *α* is the optical absorption coefficient, and *hν* is the energy of the incident photons. From these curves we can estimate the optical band gap (*E_g_*) values, by the extrapolation of straight line to ${\left(\alpha h\nu \right)}^{2}=0$. Figure 4(b) shows band gap values varying from 3.44 to 3.56 eV as a function of water content in the starting solution.

### 3.4. Electrical Results

Table 1 shows the electrical resistivity values for ZnO thin films deposited with solutions with different water contents, for fixed T_S_ = 430 °C. A minimum in the resistivity value occurs for samples deposited with a water content of 100 mL/L. Further increase in water content causes an increase in the resistivity of ZnO thin films. The variation in water content in the starting solution for the deposition of ZnO thin films leads to substantial modification in the surface morphology. We believe this can be due to the characteristics of the deposition process. Namely, as the small drops travel toward the hot substrate, gradual evaporation of solvents occurs. This in turn causes an increase in the concentration of zinc salt lowering the corresponding pH of the solution. As a result, formation of irregular shaped grains of ZnO is observed. The increase in the water content changes the growth process, as the evaporation of solvents occurs more slowly, particularly the source of oxygen, water in this case, and increasing the chance for complete ZnO formation. In this case, ZnO with hexagonal slice shaped forms are obtained. However, in the low regime water content, a wide distribution in grain size is also observed that can be explained as a result of non-uniform distribution of oxygen source on the substrate, indicative of preferential nucleation around bigger grains. Further increase in water content, above a critical value, leads to the formation of grains with almost the same size, retaining the hexagonal slice shape. This effect can be due to the uniformity of oxygen content throughout the substrate, as a super-saturation of water occurs during the growth process, enhancing uniform grain growth. Consistency in morphology changes is also observed with the corresponding electrical results, as scattering of free carriers by grain boundaries is high in a film formed by hexagonal slice shaped grains, compared with an almost continuous growth occurring in films deposited with the lowest water content. The transmittance can be affected by texturing effect due to the water content on the characteristics of ZnO:In thin solid films.

**Table 1:** The electrical resistivity and the band gap values for ZnO:In thin films deposited with solutions with different water content, for fixed TS = 430 °C. V_H2O_ = H_2_O volume; V_Total_ = Total volume of H_2_O.

ID Sample	V_H2O_ /V_Total_	Average thickness (nm) ±5.0%	Average transmittance (400–700 nm) (%)	Average transmittance (at 550 nm) (%)	Electrical Resistivity x10^-3^ ( Ωcm) ±5.0%	Band gap ( eV) ±5.0%
M1	50/1000	673	56.87	58.97	1.54	3.56
M2	100/1000	655	59.23	62.39	1.31	3.51
M3	150/1000	680	55.08	58.19	1.90	3.49
M4	200/1000	685	53.11	71.61	2.05	3.51
M5	300/1000	435	69.05	79.61	10.17	3.44

## 4. Conclusions

The role of water content on the characteristics of ZnO:In thin films has been shown. Microstructure, morphology and electrical characteristics of the films can be achieved solely by solvent composition. Optimum deposition temperature conditions were obtained at 430 °C. Some changes in the preferential growth, from random to highly oriented along the (002) planes in samples deposited at low and high temperatures, respectively, were observed. It is worth mentioning that by using the USP method, there is a great saving of reactants as compared with the pneumatic spray pyrolysis process. Based on these results, the ZnO:In thin films processed by ultrasonic spray pyrolysis show a great potential to be applied as transparent conducting electrodes in thin film solar cells with electrical conductivity close to doped In_2_O_3_, [ITO], and with appropriate high optical transmission in the near-UV, VIS and NIR. Further studies on the deposition of ZnO:In films and optimization of the implemented CST parameters should also be performed in order to improve the nonlinear optical -response of the samples and film quality.
